# A critical review of the allocentric spatial representation and its neural underpinnings: toward a network-based perspective

**DOI:** 10.3389/fnhum.2014.00803

**Published:** 2014-10-10

**Authors:** Arne D. Ekstrom, Aiden E. G. F. Arnold, Giuseppe Iaria

**Affiliations:** ^1^Center for Neuroscience, University of California at DavisDavis, CA, USA; ^2^Department of Psychology, University of California at DavisDavis, CA, USA; ^3^Neuroscience Graduate Group, University of California at DavisDavis, CA, USA; ^4^Department of Psychology, Hotchkiss Brain Institute and Alberta Children’s Hospital Research Institute, University of CalgaryCalgary, AB, Canada

**Keywords:** cognitive map, hippocampus, humans, path integration, spatial navigation, allocentric, egocentric

## Abstract

While the widely studied allocentric spatial representation holds a special status in neuroscience research, its exact nature and neural underpinnings continue to be the topic of debate, particularly in humans. Here, based on a review of human behavioral research, we argue that allocentric representations do not provide the kind of map-like, metric representation one might expect based on past theoretical work. Instead, we suggest that almost all tasks used in past studies involve a combination of egocentric and allocentric representation, complicating both the investigation of the cognitive basis of an allocentric representation and the task of identifying a brain region specifically dedicated to it. Indeed, as we discuss in detail, past studies suggest numerous brain regions important to allocentric spatial memory in addition to the hippocampus, including parahippocampal, retrosplenial, and prefrontal cortices. We thus argue that although allocentric computations will often require the hippocampus, particularly those involving extracting details across temporally specific routes, the hippocampus is not necessary for all allocentric computations. We instead suggest that a non-aggregate network process involving multiple interacting brain areas, including hippocampus and extra-hippocampal areas such as parahippocampal, retrosplenial, prefrontal, and parietal cortices, better characterizes the neural basis of spatial representation during navigation. According to this model, an allocentric representation does not emerge from the computations of a single brain region (i.e., hippocampus) nor is it readily decomposable into additive computations performed by separate brain regions. Instead, an allocentric representation emerges from computations partially shared across numerous interacting brain regions. We discuss our non-aggregate network model in light of existing data and provide several key predictions for future experiments.

Central to considering how we represent our spatial surrounding, Edward Tolman (1948) first proposed that the brain creates a “cognitive map” of a spatial environment. Based on his work primarily in rodents, Tolman linked a specific cognitive map to a certain spatial environment (analogous to a cartographic map) such that the position of an object within that environment could be derived from reference to at least two other landmarks. This perspective argued against the idea that a rodent’s representation of the surrounding environment was based solely on self-referenced (egocentric) sequences of turns, demonstrating that the internal representation of space must be more integrated and comprehensive than previously assumed by behaviorist researchers. Since Tolman, the idea that most species, including humans, posses multiple mechanisms for navigating, including one dependent on information about the position of the self relative to the environment (egocentric) and another regarding the position of other objects position relative to each other in the environment (allocentric), is generally well accepted, with some caveats we will discuss. In contrast, the exact nature of these representations, when and in what manner the two representational systems manifest and interact, and what brain areas are critical for them, particularly in humans, remains less clear. Thus, the first primary challenge we will consider is exactly how and in what manner an allocentric representation manifests during behavior and to what extent it operates exclusively, or most often, in the presence of egocentric representation.

Subsequent research has often focused on one specific brain area in particular, the hippocampus, in housing the neural machinery underlying the cognitive map. While there is overall broad consensus regarding the involvement of the hippocampus in allocentric memory, there is significantly less consensus across both empirical and theoretical studies, particularly in humans, regarding the primary (i.e., necessary) role of the hippocampus to all forms of allocentric memory. Here, we will explore some reasons why pinning down a primary role for the human hippocampus in allocentric memory across studies has been challenging, including both the difficulty of identifying “process-pure” allocentric tasks and the fact that multiple brain regions contribute necessary functions to allocentric memory. We then attempt to define a network-based model of spatial navigation addressing some of these potential short-comings.

## DEFINING AND MEASURING ALLOCENTRIC MEMORY IN HUMANS: APPROACHES AND CHALLENGES

Before we begin our discussion, it is helpful to define and clarify some of the basic assumptions and ideas we will be working with throughout. We use the term “navigation” as a proxy for the processing of a variety of different forms of information during self-movement that may lead to different cognitive strategies useful for ultimately finding our way to a given destination. This information is most often visual but also vestibular, proprioceptive, somatosensory, and auditory. Although humans have a bias toward using visual information, the others are often processed as well, and they may all contribute (either in a combined fashion or independently) to extracting information about the environment (e.g., its shape and scale), the location of items, and our own location within it. While navigating, we become familiar with the environment and acquire knowledge about it, thereby extracting information from it and storing this information in our memory so that we can recall it later for a variety of purposes. The process of extracting information from our environment can be quite rapid, particularly if we can view useful features, like landmarks, by scanning the environment (Ishikawa and Montello, 2006; Wolbers and Wiener, 2014); it can also take time, depending on the size of the environment (Siegel and White, 1975). One form of such information that we (often) begin extracting and storing is a mental representation of where things are in space with respect to each other independent of our own location (i.e., an allocentric representation; often termed a “cognitive map” due to its similarity to a cartographic map that is used in unfamiliar surroundings to access such information).

**FIGURE 1:**
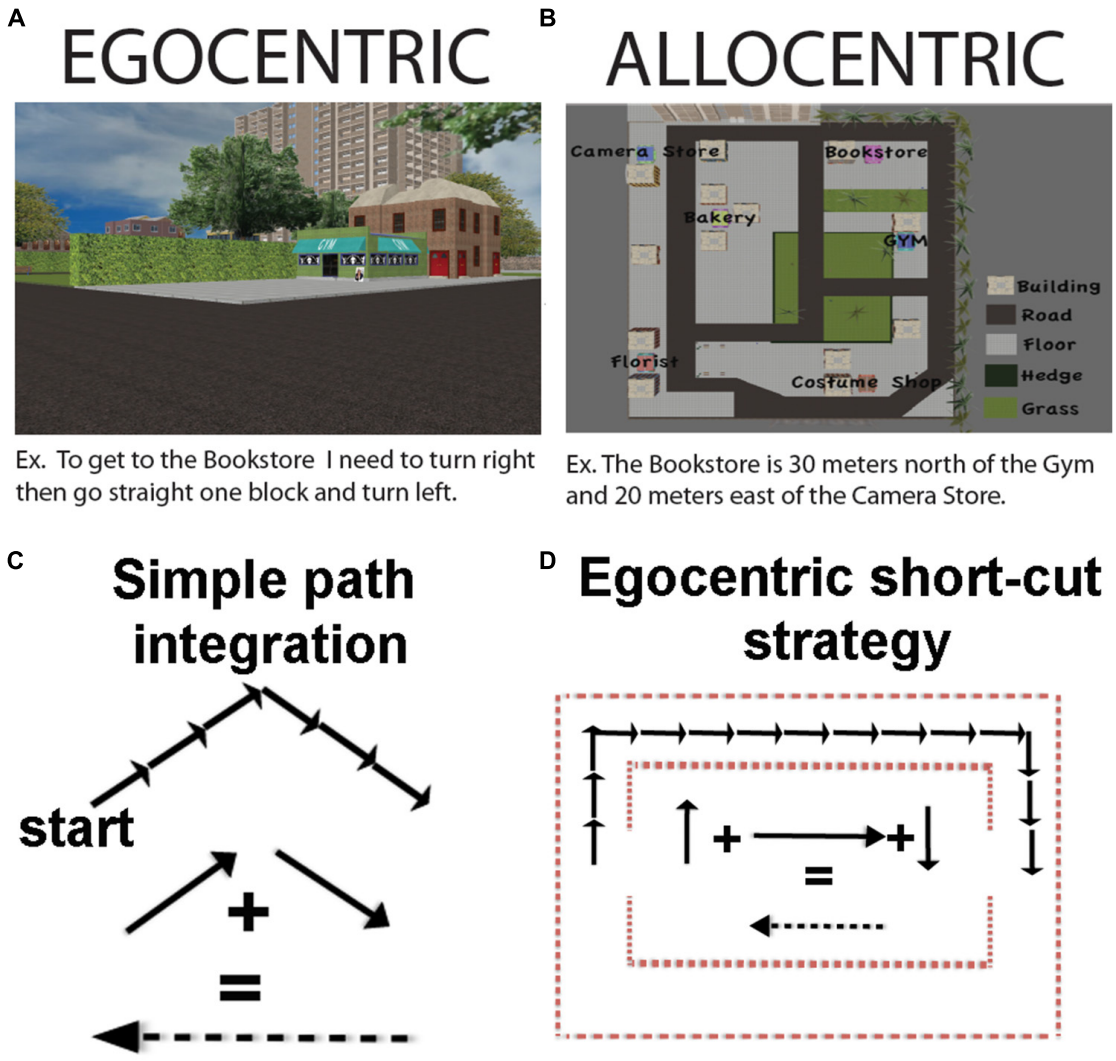
**(A)** An example of an egocentric coding strategy. **(B)** An example of an allocentric coding strategy. **(C)** How path integration can occur using a predominantly egocentric coding scheme. **(D)** How navigation short-cuts can occur using a predominantly egocentric (path integration) strategy. Note that in **(C)** and **(D)** we have flipped the sign of the resultant vector, which would equate to turning 180 degrees, but does not alter the vector quantity.

The use of an allocentric representation will most often be pronounced at decisions points, and in particular, when we make judgments about the relative position of objects based on our memory of the location where they have previously been encountered. For example, when arriving at a landmark, or viewing it from a certain distance, we could remember that our destination is positioned between this landmark and another one, sitting about 2/3 of the way from the 2nd landmark and at a 30∘ angle from the first one. Such decisions on where things are in space with respect to one another and the actual location of the individual, however, do not necessarily depend on an allocentric representation. For example, we could also remember, based on our past experience, that our goal is present 50 and 30∘ to the right of our current position, which would be an egocentric form of spatial judgment (**Figures 1A,B**; see also Wolbers and Wiener, 2014). Therefore, a mental representation of the environment can involve either allocentric or egocentric spatial representations, or most commonly, both. Whatever representation we use at decision points, however, we must be able to eventually represent the environment relative to our immediate position in space in order to select an appropriate route, make the correct turns, and travel the correct distance to our goal. Thus, for our purposes here, we consider navigation as a process that will most often involve a primarily egocentric form of representation as it depends, first and foremost, on orientation and locomotion of the individual in space.

As alluded to earlier, early research tied the idea of an allocentric representation closely to a cognitive map, which was postulated to posses many of the same qualities as a cartographic map (Tolman, 1948; O’Keefe and Nadel, 1978; Gallistel, 1990). This extended the conceptualization of an allocentric representation to the idea that these representations also involve some of the metric properties of cartographic maps themselves. Critically, this includes the idea that cognitive maps involve a fundamental Euclidean metric framework (O’Keefe and Nadel, 1978; Gallistel, 1990), suggesting that objects are accurately represented both according to their distance and angular relationships, akin to how they are in the real world (O’Keefe and Nadel, 1978). Subsequent work in humans, though, has generally not supported the idea that situations involving utilization of an allocentric representation possess the same characteristics as cartographic maps, particularly their Euclidean qualities (for a review, see: Tversky, 1992). For example, prior heuristic knowledge (Stevens and Coupe, 1978), experience with specific egocentric viewpoints (Shelton and McNamara, 2001), and geometrically prominent features (McNamara et al., 2003; Cheng and Newcombe, 2005) influence how these representations manifest.

In one widely cited and discussed example of our spatial representations differ from cartographic maps, Stevens and Coupe (1978) asked participants to indicate which cities from a list were further west. Although participants made many of these judgments correctly, one particular error occurred for decisions involving Reno and San Diego (Reno is further west due to the geography of the U.S.). Participants consistently indicated that San Diego was further west, suggesting that category heuristics (that California is further west than Nevada) overode actual metric Euclidean knowledge of maps. In another example of inaccuracies in our metric knowledge of space, several studies have shown that we systematically underestimate the Euclidean properties of spatial geometries. In one such study, both blindfolded sighted and blind participants were asked to complete a third leg of a triangle by walking it after traversing the first two legs. Participants were not very accurate at the task and often made direction and distance errors in their return path (Loomis et al., 1993; for a detailed discussion, please see: Philbeck et al., 2001). While the triangle completion task can be solved using a primarily egocentric updating strategy, which we will discuss in more detail shortly, others have argued that path integration involves interaction with a cognitive map (Gallistel, 1990; McNaughton et al., 1991), thus relying, in part, on allocentric coding. From this perspective, errors in the triangle completion task indicate that our internal “map” of space often does not mirror the metric properties of physical space. Together, these findings demonstrate our spatial judgments are subject to systematic distortions and errors, suggesting that our cognitive map, at least with limited exposure, is not comparable to an actual cartographic map.

Another factor that appears to influence how we utilize an allocentric representation is our experience with current and prior viewpoints (Sholl, 1987). In one of many examples of this, Sholl (1987) tested participants on their knowledge of locations of campus buildings (see also: Werner and Schmidt, 1999; Frankenstein et al., 2012). Participants were oriented in the room relative to campus and told to point to campus landmarks. Participants were significantly faster to identify campus landmarks located in front of them vs. behind them. Thus, although participants had knowledge of the relative position of landmarks on campus, pointing accurately in most cases, there was a clear advantage for allocentric information oriented with their current bearing. In a similar vein, several studies have also suggested an advantage for retrieving allocentric information consistent with the viewpoint from which it was originally encoded. Specifically, if participants learn an array of objects arranged in a room, they are significantly faster and more accurate at retrieving their location in the absence of the objects if their viewpoint is aligned with what they originally experienced (relative to the axes in the room) compared to a misaligned viewpoint (Diwadkar and McNamara, 1997). The same orientation-dependent properties hold for judgments about information learned from large scale environments (Roskos-Ewoldsen et al., 1998), cartographic maps (Evans and Pezdek, 1980), and for navigation in virtual environments (VE; Richardson et al., 1999). Thus, for both allocentric spatial relationships learned by viewing them or freely navigating, orientation biases how we store and retrieve these representations.

Another form of navigation sometimes taken as an example of allocentric representation is path integration (McNaughton et al., 1991). Path integration is a situation in which a participant produces a novel path on having completed two (or more) other components of the journey. One particularly well-known example of path integration is when a gerbil pup is separated from its mother. The mother will forage with a wandering path until she finds her pup. Once the pup is found, however, she will take a direct route to get the pup back to safety in the nest (Mittelstaedt and Mittelstaedt, 1980). In humans, path integration has frequently been studied by having participants walk the first two legs of a triangle and then determine the optimal path, or “short-cut,” back to the origin. While on the surface it might seem that path integration would necessitate map-like knowledge of the environment (Tolman, 1948; O’Keefe and Nadel, 1978; McNaughton et al., 1991), this is not always the case. As was subsequently pointed out in later work, path integration can also be accomplished using an egocentric updating strategy (keeping track of one’s bearing and distance and comparing with the bearing of the start position; Wang and Spelke, 2002). As alluded to earlier, this can operate, in principle, with no external landmarks, and thus no need for allocentric representation (**Figure 1C**).

In practice though, at least in humans, path integration in environments without landmarks involves substantial errors (Philbeck et al., 1997; Foo et al., 2005) and eventual complete disorientation at long enough distances, such as in the desert (Souman et al., 2009). While path integration in humans is comparably more accurate when ample landmarks are provided (Foo et al., 2005), whether this involves additional allocentric computations based on generalizing from egocentric position cues provided by the landmark remains unclear. Thus, employing short-cuts and/or using path integration strategies is not necessarily indicative of using an allocentric strategy as it can be accomplished by employing primarily egocentric cues. In **Figure 1D**, we illustrate an example of how short-cuts could potentially be solved using a primarily egocentric form of representation. The importance of landmarks to path integration, however, suggests it is not a purely egocentric strategy either. To be clear, employing short-cuts does not work *necessarily* at the exclusion of egocentric or allocentric representation; for the reasons outlined above, it cannot be considered a purely, or even primarily, allocentric or egocentric task, either.

A final issue we will mention here is an important point regarding the scale of navigational space considered in the task. As elegantly pointed out in a recent review by Wolbers and Wiener (2014), many tasks appearing to involve allocentric representation, such as the well-known Morris Water Maze (Morris et al., 1982), also involve judgments in relatively small scale space, which they term “vista space.” This suggests, though, that such tasks can often be solved using information that can be captured with a single viewpoint (see also: Yamamoto and Shelton, 2009). While viewpoints can still involve use of either allocentric or egocentric forms of representation, depending on whether or not there is reference to external landmarks (Wolbers and Wiener, 2014), the majority of control conditions involving the Morris Water don’t involve a clear need for egocentric representation (representation of the bearing of landmarks relative to oneself). Instead, they can be based on simple visuo-motor strategies or viewpoint matching. Even an allocentric representation in vista space, such as reference to two (or more) external landmarks, can involve a single snapshot (Wolbers and Wiener, 2014), which could in principle be rotated by body repositioning to be solved egocentrically (Simons and Wang, 1998). Thus, in our discussion of allocentric representation, it is important to consider both the scale of space, as well as the extent to which alternative egocentric strategies might permit a solution to what otherwise appears as a primarily allocentric task.

## WHEN *DON’T* EGOCENTRIC AND ALLOCENTRIC REPRESENTATIONS COEXIST AND INTERACT: HOW TO DETERMINE THE PRESENCE OF EGOCENTRIC AND ALLOCENTRIC REPRESENTATION

The above perspective necessitates an important question: to what extent might one observe a process “pure” allocentric task? It has been argued in some reviews that egocentric coding schemes dominate in most human spatial memory studies, such that the only cases in which one might observe an allocentric form of representation is based on the geometry of the room (Wang and Spelke, 2000, 2002). In one such study demonstrating the dominance of egocentric viewpoints, the authors had participants learn the locations of objects in a room. Participants were then blindfolded and either slowly rotated or rotated quickly to induce disorientation. While knowledge of the positions of objects in the room remained high when participants remained oriented, disorientation resulted in almost complete loss of knowledge of locations of objects in the room. Even following disorientation, however, participants could accurately point to corners of the room (particularly in terms of their relative locations). These findings argued that in the absence of orienting cues, which strongly influence egocentric representation, our knowledge of allocentric relationships drop precipitously, with the exception of the boundaries defined by the room (but see: Holmes and Sholl, 2005).

**FIGURE 2:**
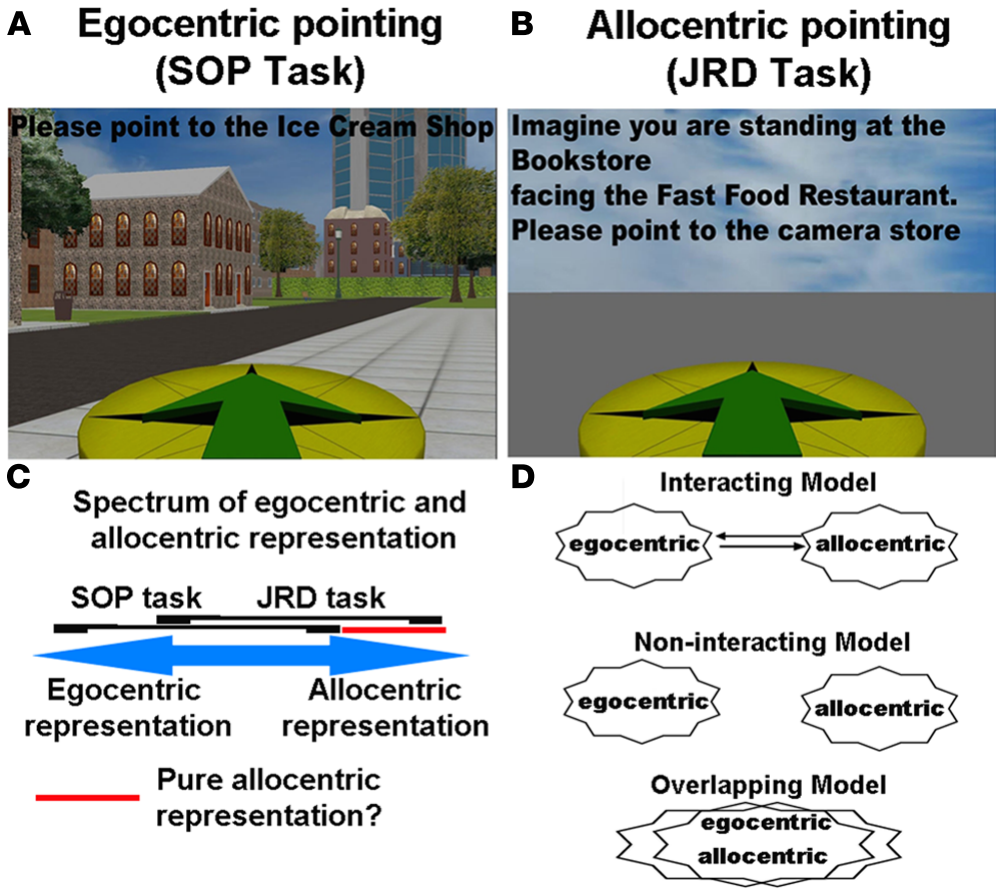
**(A)** The scene and orientation-dependent pointing (SOP) task, often employed to assay an egocentric representation. **(B)** The judgments of relative direction (JRD) task, often used to assay an allocentric representation. **(C)** Spectrum of involvement of egocentric and allocentric representation during the SOP and JRD tasks. **(D)** Different models in the literature of how egocentric and allocentric representations may be coded.

There are two important points, however, about the Wang and Spelke (2000) study. The first regards the measure that Wang and Spelke used, which we term here the scene and orientation dependent pointing (SOP) task. This task involves participants simply pointing to objects based on their current location (i.e., point to the computer monitor, **Figure 2A**), either when blindfolded or with the targets removed. A subsequent study by Waller and Hodgson (2006), using a similar paradigm to Wang and Spelke, in contrast, showed that during judgments of relative direction (JRD), which involved reference to external landmarks, pointing accuracy actually improved following disorientation while SOP accuracy dropped (Waller and Hodgson, 2006). In the JRD pointing task, participants made reference to least two other objects when pointing to a third (e.g., imagine you are standing at the flower pot, facing the jar, now point to the coffee mug; see **Figure 2B**). Thus, when asking participants to respond using the relative positions of objects within the environment, pointing accuracy actually improved. This double dissociation suggested that part of what may happen to disoriented participants is that when asked to rely on a task involving their current orientation (like the SOP task), they are unable to access any allocentric knowledge. In contrast, when solving a task like the JRD task, which explicitly asks participants to think in terms of spatial relationships of recently learned objects, they employ a more allocentric-based strategy (Burgess, 2006). Thus, these data in fact suggest that participants can utilize allocentric knowledge, but when and how they do so depends to some extent on how they are queried.

The second issue involves the testing set-up used in the Wang and Spelke (2000) study. It is notable that many studies of human spatial memory involve learning arrays of objects in relatively small-sized, regularly shaped rooms over a single trial of learning. As pointed out above, though, if we consider larger scale space (termed by Wolbers and Wiener “environmental space”) rather than vista space, utilization of an allocentric representation might be more obvious since viewpoints cannot readily be used to solve the task. In these situations, however, given the complexity of the environment to be learned, it may take several trials for different forms of spatial knowledge to manifest. In one such study in which we addressed this issue, participants learned large virtual spatial layouts (∼300 square meters) either by directly navigating it or learning it from a map. Testing a total of five blocks of navigation and map learning interspersed with either the SOP or JRD task, we found differential improvements in SOP and JRD pointing accuracy depending on whether participants learned the environment from a route or cartographic map. Specifically, map learning resulted in the fastest improvements in JRD pointing accuracy while route learning resulted in the fastest gains in SOP pointing accuracy. Importantly, map learning resulted in fast, non-linear improvements in JRD pointing accuracy compared to route learning (Zhang et al., 2014). Together, these data suggest that while both learning modalities affect putative egocentric (measured via the SOP task) and allocentric (measured via the JRD) knowledge, route learning provides preferential access to egocentric knowledge and map learning provides preferential access to allocentric knowledge. Our study also suggests, however, that both egocentric and allocentric forms of representations are typically involved in spatial learning, consistent with previous behavioral studies in humans (Mou et al., 2004; Waller and Hodgson, 2006).

An important caveat, however, which is generally recognized in the human spatial cognition literature and we alluded to in our definitions section, is exactly how to measure egocentric and allocentric forms of representation in the first place. For example, the SOP task involves participants pointing to unseen targets based on their current orientation. This does not preclude, however, that participants also utilize some form of allocentric knowledge. Specifically, participants could easily picture their current position on a map and point to objects based on their knowledge of their position relative to other positions of objects in an environment, treating themselves no differently than other objects in the environment. While the SOP task certainly does not preclude employing allocentric knowledge of objects in the environment, however, the fact that pointing accuracy falls so drastically during disorientation (Wang and Spelke, 2000; Waller and Hodgson, 2006) seems to argue against this idea somewhat. By the same token, when participants perform the JRD pointing task, which involves imaging oneself at a position in the environment, they must first orient themselves within the environment, which would seem to utilize some degree of egocentric positioning information. While the use of orientation cues can be mitigated somewhat by having participants picture novel (rather than familiar) positions and orientations in the environment, it is clear that orientation provides some benefit in the task. At the same time, without some knowledge of the spatial relationships between objects in the environment, it seems unlikely that participants could solve the task using only orientation cues. In **Figure 2C**, we outline the extent to which egocentric and allocentric representation might, in theory, overlap with the SOP and JRD tasks. As is notable in **Figure 2C**, we suggest a large area of overlap between the two tasks, with some areas of non-overlap. This indicates that while both tasks involve some form of the two representations, each will involve some predominance of egocentric and allocentric representation, respectively.

## EGOCENTRIC TO ALLOCENTRIC CONVERSION AND VICE VERSA: THE ABSENCE OF PURE FORMS OF EGOCENTRIC AND ALLOCENTRIC REPRESENTATION

As pointed out in the above arguments and **Figure 2C**, even tasks widely used in human spatial learning to assess egocentric and allocentric knowledge likely do not tap into one form of representation exclusively. In fact, most ethological situations would appear to involve some combination of two forms of representations, with a spectrum of when the two might dominate perhaps being the most accurate way to conceptualize how we represent a spatial environment (**Figure 2C**). While the farthest end of this spectrum might involve a “pure” form of allocentric representation, there is currently no clear situation in which a participant might use an allocentric representation in the complete absence of any egocentric representation. A “pure” allocentric representation might be expected to be present, at least cognitively, when participants make judgments about the positions of landmarks relative to each other (without reference to self-position). Because this “pure” form of allocentric representation is hard to isolate experimentally, it remains difficult to validate.

One interesting proposal that provides a possible solution to the above problems regarding the relative absence of a “pure” allocentric representation is provided by the influential Byrne et al. (2007) model of spatial learning (which we term the BBB model). The BBB model argues that almost all situations require translation between egocentric and allocentric representation, suggesting that most situations would likely involve a mixture of the two forms of representation (**Figure 2D**, “interacting model”). The crux of the model argues that egocentric representations are primarily housed in parietal cortex, representing information of one’s current viewpoint based on one’s current position and bearing (Byrne et al., 2007). Allocentric representations are housed in the medial temporal lobe (MTL) and involve place cells, neurons that code specific spatial locations and thus provide some degree of metric knowledge about locations in the environment (O’Keefe and Dostrovsky, 1971). Finally, retrosplenial cortex, a brain area that sends and receives projections from parietal cortex and sends and receives projections to the MTL, performs translation between egocentric and allocentric coordinate frames. In the model, this involves aligning the current “map” in the MTL with one’s current viewpoint such that a view specific map is computed in retrosplenial cortex. This would be akin to a map that is aligned based on our current bearing, so if we are facing east, it would be like orienting the map eastward. In addition to the neuroanatomical considerations we will consider shortly, this model thus provides an explanation of why most situations would preclude us from observing “pure” allocentric representations, as retrosplenial cortex would frequently “align” whatever map-like knowledge we have with our current bearing. Thus, the model makes the important prediction that in most situations, neither pure egocentric nor allocentric knowledge will necessarily be at play because some degree of conversion between the two forms of representation will occur during navigation.

An important alternative we might consider here is the idea that there is no single egocentric or allocentric representation but instead that all forms of representation involve essentially a hybrid between the two (Sheth and Shimojo, 2004). This counter point thus would argue (1) egocentric to allocentric representation is unnecessary because the two representations are really one “combined” representation (**Figure 2D**), (2) brain damage is unlikely to selectively disable either form of representation because both are present in multiple brain systems, and (3) brain imaging will likely fail to identify one region clearly associated with either form of representation, or conversion process, instead showing that both forms of representation involve a mixture of brain areas. This argument is weakened, however, by the fact that lesion and neuroimaging studies suggest some distinct regions participate in tasks involving primarily egocentric vs. allocentric strategies, as we will explore in more detail shortly. The behavioral double dissociations reported by Waller and Hodgson and Zhang et al. (2014) for SOP and JRD pointing tasks also argues against this model (Waller and Hodgson, 2006; Zhang et al., 2014). To be clear, though, showing that one region is more involved in one form of representation than another does not preclude the fact that the other representation is also partly at play (nor does a behavioral double dissociation necessarily exclude this possibility). It only suggests that in many situations, either a primarily allocentric or egocentric strategy is the preferred or simpler way to solve the task, leaving behavior comparatively impaired when relying on the other representational system.

A final point, which we will consider in more detail shortly, is that even if we could identify a process-pure allocentric task, no single brain region serves as the primary neural underpinning for what manifests in behavior as use of an allocentric strategy. This argument is more complex and essentially involves the idea that (some) higher cognitive functions cannot readily be decomposed into the contributions of a single brain region. Thus, it might be that employing an allocentric strategy to solve a task, which we infer involves an allocentric representation, recruits a network of different brain regions, with no single process or brain region contributing a unique, separable process in the form of an allocentric representation. This account would still allow for the idea that lesions to brain “hubs” within this network would be disruptive to solving a task allocentrically but would not require that a single brain region is necessary for all forms of tasks involving an allocentric navigation strategy. We will explore this argument in more detail shortly.

## BRAIN IMAGING AND LESION STUDIES: THE NEURAL BASIS OF ALLOCENTRIC REPRESENTATION

As we have argued so far, different tasks likely involve different mixtures of egocentric and allocentric representation. In fact, individuals may differ even in the extent to which they employ one form of representation over another while navigating (Marchette et al., 2011). We believe that these may be possible reasons why past literature has been ambiguous, particularly in humans, regarding the neural basis of allocentric representation. Nonetheless, we should make clear, before talking about some of the inconsistent findings in the literature, that many studies have in fact attributed a primary role to the hippocampus in allocentric memory. For example, a classic study from the rodent literature, which has been replicated numerous times (for a review, see: D’Hooge and De Deyn, 2001), showed that lesions to the rat hippocampus impaired its ability to use distal cues to find the goal in the Morris Water Maze (Morris et al., 1982). Specifically, lesions to the rat hippocampus impaired its ability to find a hidden platform over learning trials and its ability to recall its location on subsequent “probe trials.” These same lesions did not affect the ability of the rodent to find the hidden platform if a brightly colored cue is placed above it (termed a “proximal cue.”) These findings have often been taken to support a fundamental role for the rodent hippocampus in map-based, allocentric-based navigation but not egocentric-based navigation (e.g., Nadel, 1991). Studies involving human patients with lesions restricted to the hippocampus show similar impairments on virtual and real versions of the Morris Water Maze, suggesting that performance of this task relies on the hippocampus in both rats and humans (Astur et al., 2002; Bartsch et al., 2010; Goodrich-Hunsaker et al., 2010; Banta-Lavenex et al., 2014).

We have already discussed some of the possible limitations with considering the Morris Water Maze as the gold standard of allocentric representation, specifically relating to the fact that the task can be solved based on manipulating a single viewpoint (Wolbers and Wiener, 2014). Furthermore, some studies have demonstrated instances in which rats with hippocampal damage can solve the Morris Water Maze. Perhaps the clearest examples of these involve rats that receive extensive pre-training on complex environments; following a hippocampal lesion, these rats are relatively unimpaired at navigating the Morris Water Maze (Winocur et al., 2005, 2010). Similar findings have also been reported from humans with hippocampal lesions, who appear to have largely intact allocentric memory for spatial layouts experienced decades prior to their brain injury (Teng and Squire, 1999; Rosenbaum et al., 2000).

Modifications to the Morris Water Maze paradigm also suggest situations in which allocentric memory may be preserved even at short delays. Day et al. (1999) showed that by gradually decreasing the size of the hidden platform in the Morris Water maze from a very large one to a small one, rats without a hippocampus could learn the location of the hidden platform. This finding suggests that the hippocampus is not necessary for acquiring or expressing allocentric knowledge in some cases. Pearce et al. (1998) used a modified version of the Morris Water Maze involving a local landmark; the hidden platform was placed within 20 cm of a local, visible landmark. When Pearce et al. (1998) moved the hidden platform to the opposite side of the local landmark, both groups of rats searched where the platform was previously and had difficulty in finding the locations of the switched platform. While it is possible this was due to using a heading strategy to solve the task (i.e., locate the visible landmark and swim toward one of the distal cues), it is also possible that rats with hippocampal lesions used some combination of local and distal cues to guide their path in the first place, thus resulting in a tendency to search in the previous location, like control rats. Otherwise, if rats were simply swimming to the local cue and using a random search strategy, one might have expected that they would have found the new location with relative ease. Note that because the rats approached the local cue from different start locations, a purely egocentric strategy (i.e., turn right at the local cue) would not work for finding the distal cue once the rat swam to the local cue. Together, these data suggest that there may be instances in which some forms of allocentric representation may be intact in rats with hippocampal lesions.

Evidence from humans also suggests that some forms of allocentric spatial memory can develop independently of the hippocampus, even at short delays. Numerous functional magnetic resonance imaging (fMRI) studies contrasting primarily allocentric with egocentric forms of navigation report parahippocampal cortex (PHC), rather than hippocampal, activation (Aguirre et al., 1996; Aguirre and D’Esposito, 1997; Committeri et al., 2004; Janzen and van Turennout, 2004; Rosenbaum et al., 2004; Wolbers and Buchel, 2005; Zhang and Ekstrom, 2013). For example, Aguirre et al. (1996) demonstrated greater PHC, but not hippocampal activation, when participants learned a maze-like VE and subsequently drew maps compared to a control task in which they walked in a constrained path around a different VE. Committeri et al. (2004) reported similar results for making judgments about static spatial scenes with reference to other landmarks vs. based on the observer’s viewpoint. While there are issues to consider regarding the ability to target a relatively small structure such as the hippocampus that is often prone to signal distortion, signal loss, and intersubject mis-registration (Fried, 1998; Yassa and Stark, 2009; Ekstrom, 2010), some of these same studies (Wolbers and Buchel, 2005; Zhang and Ekstrom, 2013) noted hippocampal activation in other contrasts. This suggests that the presence of PHC activation and absence of hippocampal activation in the above fMRI studies using contrasts likely to tap into allocentric processing were unlikely to have arisen from imaging-related methodological issues alone. These findings support the idea that the PHC may be involved in some situations involving allocentric computations when the hippocampus is not.

Indeed, lesions to the PHC appear to impair some forms of spatial navigation involving allocentric memory in situations in which hippocampal lesions do not. Bohbot et al. (1998) tested lesion patients on the invisible sensor task, a real-world analog of the Morris Water Maze in which participants explored a room searching for a hidden sensor on the floor (Bohbot et al., 1998). Lesions were either primarily restricted to the hippocampus or extended into the PHC. In the task, patients entered the room from the opposite entrance on the first trial of retrieval, which occurred immediately after encoding, and then from the same entrance as during encoding 30 min later. Bohbot et al. (1998) found that right PHC lesions impaired spatial learning but only after a 30-min delay was implemented, whereas damage to the left or right hippocampus, with relatively spared PHC, produced normal performance on both immediate and delay tasks. Consistent with other lesion studies in the literature, the Bohbot et al. (1998) study supports the idea that the PHC could be important for tasks that might otherwise seem more readily solved using a primarily allocentric strategy (Habib and Sirigu, 1987).

## PARAHIPPOCAMPAL AND RETROSPLENIAL CORTEX AS LOCI OF NEURAL MACHINERY IMPORTANT TO SPATIAL REPRESENTATION AND NAVIGATION

As we discussed above, PHC lesions, in many instances, disrupt the ability of patients to navigate to recently learned locations (Bohbot et al., 1998; Barrash et al., 2000). Given our earlier discussion, however, regarding the fact that almost all ethological conditions involve some mixture of egocentric and allocentric strategy, it seems reasonable to consider that the patients in the Bohbot et al. (1998) study may have employed an egocentric strategy. While patients did enter from the same door during retrieval after the 30 minute delay in the study, which may have permitted use of a strategy involving matching of viewpoints, subsequent analysis of the path taken by the participants suggests they were not simply matching their previously stored viewpoint with their navigational trajectory (Bohbot et al., 2002). If they were, we would have expected that participants would have navigated directly to the goal from the door. It is possible, however, that patients with hippocampal lesions may have employed a partially egocentric strategy nonetheless, and based on our earlier discussion, this idea is difficult to completely rule out without follow-up experiments. As mentioned above, however, numerous fMRI studies have shown activation in the PHC during retrieval of spatial layout information. Indeed, one of the most highly replicated studies in the fMRI of spatial processing is the fact that the parts of posterior parahippocampal gyrus [termed the parahippocampal place area (PPA)] respond particularly robustly to scenes (Epstein and Kanwisher, 1998) and respond differently to scenes from different viewpoints (Epstein et al., 2003). While this has been taken by some researchers to support the idea that the PHC is important for egocentric processing (Weniger et al., 2010), other studies have suggested that areas of PHC show greater degrees of activation when participants make judgments about scenes based on reference to other objects in the scene rather than to themselves (Committeri et al., 2004; Zhang and Ekstrom, 2013).

Additional evidence supporting the role of PPA in spatial scene processing comes from an analysis technique termed multivariate voxel pattern analysis (MVPA). In this approach, patterns of voxels are used to predict the stimulus that a participant viewed (called the test set) based on a prior set of similar stimuli they viewed (called the training set). This approach consistently indicates that spatial scenes can be readily decoded from PPA and retrosplenial cortex, although less readily from the hippocampus (Diana et al., 2008; Liang et al., 2013; see also: Azab et al., 2014). Thus, together, these results suggest that PHC shows a high degree of sensitivity to the visual content of scenes compared to the hippocampus.

Retrosplenial cortex also shows strong responses to spatial scenes and spatial features, as measured with the fMRI blood oxygen level-dependent (BOLD) signal (Epstein and Higgins, 2007; Auger and Maguire, 2013; Zhang and Ekstrom, 2013). Furthermore, lesions to retrosplenial cortex significantly impair the ability of participants to change their view perspective (Takahashi et al., 1997). As proposed by Burgess et al. (2001), retrosplenial cortex likely plays a role in combining head direction information from anterior thalamic nuclei to with the current viewpoint and stored scene representations, what can also be termed egocentric to allocentric conversion. One of the first lines of evidence in support of this idea came from Iaria et al. (2007), who demonstrated engagement of retrosplenial cortex while individuals both acquired knowledge of a new environment and when making use of it to find a target location. Based on these findings, the authors suggested that the role of the retrosplenial cortex may involve both egocentric–allocentric conversion as well as an allocentric–egocentric one. Several studies have additionally shown that the BOLD signal in retrosplenial cortex is correlated with the amount of allocentric knowledge acquired following learning the spatial relations in an environment, suggesting its involvement in extrapolating from largely egocentric-based route information to allocentric-based maps (Wolbers and Buchel, 2005; Epstein et al., 2007). Also, when participants make allocentric spatial judgments following route learning, they show greater retrosplenial activation than following cartographic map learning (Zhang et al., 2012). These data again support the idea that retrosplenial cortex is involved in processes that place the observer in the large-scale representation of the surrounding space by incorporating current view with self-motion cues.

## WHY ARE HIPPOCAMPAL CONTRIBUTIONS TO ALLOCENTRIC MEMORY IMPORTANT?

One proposal to reconcile the conflicting findings on hippocampal vs. PHC/retrosplenial contributions to allocentric spatial memory is that hippocampal contributions may be most evident when spatial information must be held across different temporal intervals (Poucet, 1993), a process termed spatiotemporal binding (e.g., Copara et al., 2014). Indeed, many situations that appear to involve allocentric memory, such as remembering spatial locations and integrating this information across multiple trials, would involve just this. In contrast, when sufficient information is presented, particularly across the same repeated trials, areas such as the PHC can support allocentric memory (Moscovitch et al., 2005, 2006), also termed “simple allocentric memory” (Burgess et al., 2002). In support of this idea, Zhang and Ekstrom (2013) employed fMRI in healthy participants while they learned the locations of stores relative to a central landmark from an aerial view of a virtual city; each store appeared on a different trial but always appeared with a centrally located landmark. During fMRI acquisition, participants navigated to a goal store starting from either the central landmark or from one of the other stores. Navigating to the target store using the central landmark resulted in PHC and retrosplenial activation, while employing one of the stores to infer the location of another store, which had never before occurred on the same trial, resulted in hippocampal activation. Similarly, Bohbot and Corkin (2007) studied place learning in the patient HM, who had damage to his anterior hippocampus but relatively intact posterior parahippocampal gyrus, using the invisible sensor task. Results showed that HM could navigate to the location of one of the hidden sensors quite well, as in Bohbot et al. (1998), but when multiple hidden sensors locations were employed across trials, performance fell to chance. These studies suggest that the PHC may support some forms of simple or “rigid” forms of allocentric spatial memory (Burgess et al., 2002; Moscovitch et al., 2006) with the hippocampus contributing in instances that require spatiotemporal integration, such as remembering or inferring different locations across trials.

## ALLOCENTRIC REPRESENTATION AS A NETWORK PHENOMENON RATHER THAN SPECIALIZED TO A SINGLE BRAIN REGION

**FIGURE 3:**
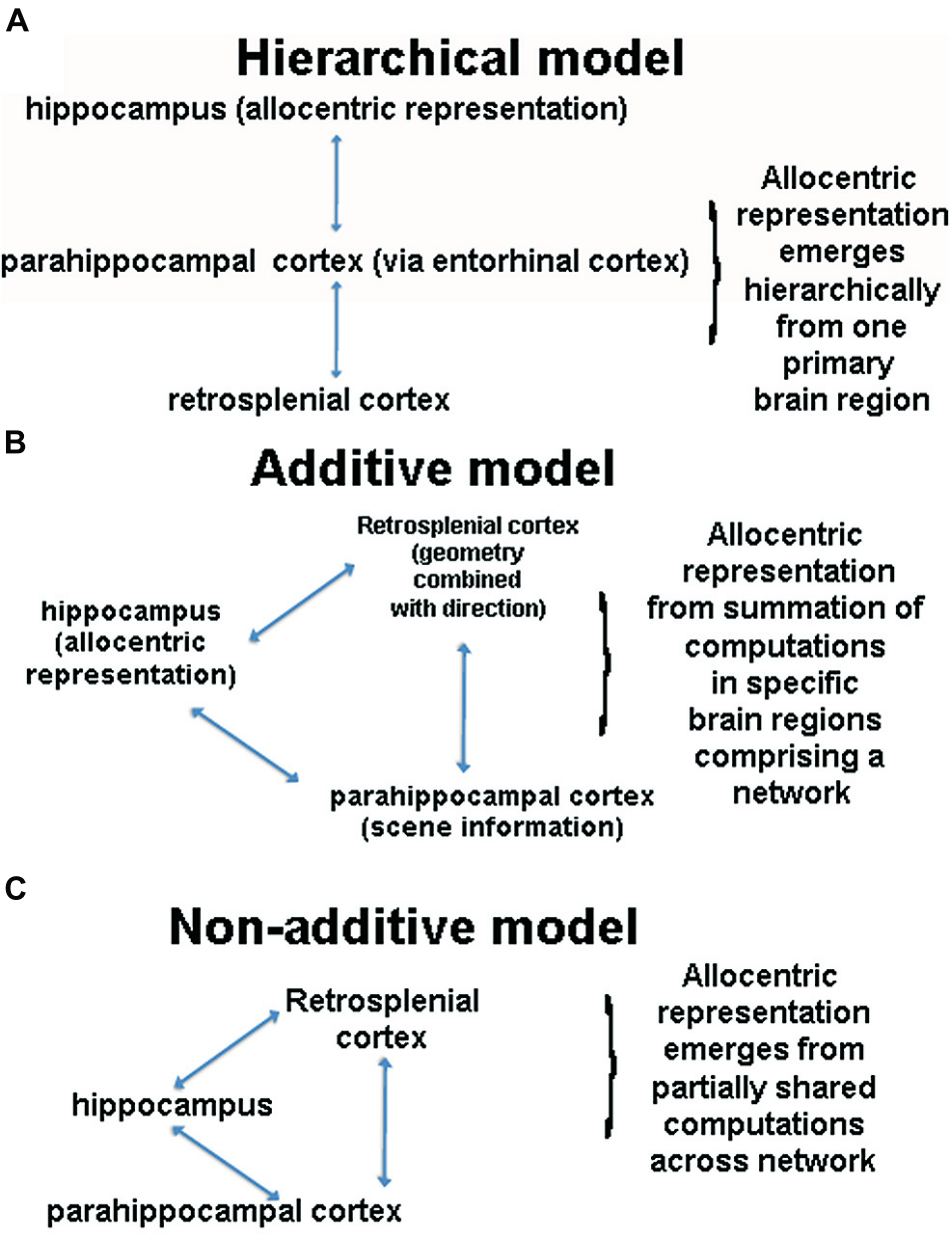
**(A)** Schematic of the hierarchical model. This model assumes that the hippocampus sits at the top of a pyramid of allocentric computations and is thus necessary for all forms of allocentric processing. **(B)** A schematic of an aggregate network model of spatial cognition. This model argues that that multiple brain regions interact during allocentric representation, with each brain region adding a unique component, the summation of which constitutes an allocentric representation. **(C)** A schematic of the non-additive network model, in which multiple brain regions support allocentric representation. Note that other brain regions and cognitive processes contribute in these models but for illustration purposes, we have focused on three primary regions.

We have summarized two fundamental yet differing perspectives so far regarding the neural basis of allocentric representation, which are depicted in **Figures 3A,B**. The first perspective is that an allocentric representation depends primarily on a single brain region, with most past studies focusing on the hippocampus as this structure. This perspective, based in large part on considerations of MTL neuroanatomy, argues that the hippocampus sits at the top of a processing pyramid and thus plays the primary role in allocentric representation (Lavenex and Amaral, 2000; Banta-Lavenex et al., 2014); see **Figure 3A**. The primary prediction from this model is that the hippocampus is necessary for all forms of allocentric representation. A second perspective, articulated in various forms in the literature, is that the hippocampus is one of several structures involved in spatial processing but actively interacts with other structures to construct an allocentric representation (Burgess et al., 2001; Byrne et al., 2007; Galati et al., 2010; Chrastil, 2012); see **Figure 3B**. This perspective suggests that the hippocampus is part of a network of other brain areas involved in allocentric representation, with each region adding a specific component to this process (Byrne et al., 2007). As we have attempted to argue so far, we believe that current data argue against the hierarchical model, providing stronger support for a network-based model. Although the network-based model still depends primarily on the hippocampus for allocentric representation, it suggests that other cortical areas, such retrosplenial cortex, add important components to this function, explaining why lesions to this area impair forms of allocentric processing dependent on bearing (Takahashi et al., 1997; Byrne et al., 2007). This model also elegantly accounts for the fact that most ethological situations involve a combination of egocentric and allocentric representation, precisely because retrosplenial cortex converts between the two representations. Thus, we believe that there is strong merit to what we term the additive network perspective and key predictions of this model remain to be tested.

A third perspective, which, to our knowledge, has not been explicitly articulated in the literature before, is the idea that multiple brain areas contribute in a non-additive fashion to allocentric representation (**Figure 3C**). The core of this idea is that no one brain region is specialized for allocentric representation and no one brain structure contributes a specific, separable “cognitive” component an allocentric representation. We note that this situation could occur if we think of an allocentric representation itself as not decomposable into elemental entities (e.g., Fodor, 1983). This, in turn, could arise because, as we have argued earlier, there are no clear situations in which we can measure a “pure” allocentic representation. As many have pointed out, though, it is reasonable to consider an allocentric representation as decomposable into landmark, direction, and distance information (e.g., Gallistel, 1990), and at least in this sense, an allocentric representation appears behaviorally decomposable. Instead what we are arguing is that even if we can decompose an allocentric representation into subcomponents, the brain areas involved in spatial cognition do not individually represent this information uniquely in a way that summates into an allocentric representation. We should be clear that the aggregate network perspective may yet be fully valid and requires further testing. It is useful, however, to consider an alternative viewpoint, which necessitates first identifying how allocentric information may come to be represented through system dynamics.

As argued in past work, it is possible to consider the functional outputs of a system along a spectrum, with one end representing aggregate (i.e., additive) functions and the other representing emergent (i.e., non-decomposable) functions. Along with other attempts to explain cognitive processes in terms of complex systems theory (Bassett and Gazzaniga, 2011), we propose that the allocentric representation sits in the middle of this spectrum and is an example of a complex neurocognitive process which is decomposable into dynamical network properties, but also non-additive in nature. An aggregate model would suggest that each brain region has its own specialized cognitive function and each brain regions “adds” this function separately to the behavior generated through the dynamics of the system (Friston et al., 1996; Friston and Price, 2011). This would predict that the allocentric representation might depend on a single brain region, or unique components contributed by individual brain regions, and thus lesions to specific brain regions should consistently and unambiguously impair tasks involving any form of allocentric representation. As argued by Bassett and Gazzaniga (2011), however, attributing single cognitive functions to separate brain regions and aggregating them together may underestimate the dynamical processes at play in the brain. A non-additive model would involve integration of processes across spatially and temporally distributed brain networks, which we believe could better capture precisely how the emergence of an allocentric representation during behavior is coupled to neural processes. According to the non-additive account, then, no single brain region contributes to either egocentric or allocentric representation independently and thus allocentric representation is a non-additive, dynamic property generated though interactions between multiple brain regions.

Although this is a new and emerging idea within the literature, and more evidence is needed to validate this perspective, we believe that the difficulties in pinning down a single brain region underlying allocentric representation, and its dynamic nature across tasks, supports this perspective. One early study supporting this perspective, although it did not use connectivity or network analyses specifically, was a study by Spiers and Maguire (2006). The authors examined taxi-drivers as they planned routes to novel destinations in a virtual version of London (Spiers and Maguire, 2006). During route planning, which one might expect to involve some allocentric computations (as well as egocentric ones), the authors found activation in a number of spatially distributed brain regions, including hippocampus, retrosplenial cortex, and prefrontal cortex (PFC). Also in support of the idea that multiple brain regions code information relevant to allocentric coding, Sulpizio et al. (2014) recently showed that position, direction, and distance could be decoded from hippocampus, PHC, and retrosplenial cortex in participants freely navigating. These findings suggest that utilizing an allocentric representation, which would be expected, in part, when executing or mentally planning a route to a location, recruits a network of different brain areas and may not be decomposable into the contributions of a single brain region or behavioral process.

Similarly, a study by Watrous et al. (2013) used functional connectivity, measured with oscillatory coherence and graph theory, to characterize network dynamics during retrieving spatial information about the relative distance between environmental landmarks. Watrous et al. (2013) found that frequency specific (1–4 Hz) increases in pairwise phase consistency correlated with retrieval accuracy within a network distributed across the PFC, MTL, and parietal cortex. In contrast, correct retrieval of temporal information about the task correlated with increased functional connectivity in a different frequency band (7–10 Hz) within a similar network (Watrous et al., 2013). In a separate study using fMRI, Schedlbauer et al. (2014) showed that greater connectivity to multiple brain “hubs” including the hippocampus correlated with overall better participant spatiotemporal memory. Together, these findings suggested that the integrative dynamics of the spatially distributed network, rather than the activity of a single region alone, was critical for accurate recall. In another study, the capacity of a similarly distributed network to integrate information was regressed against accuracy in using allocentric information to orient within a virtual city (Arnold et al., 2014). The authors found that the global efficiency [i.e., a graph theoretical index of integrative capacity, see Bullmore and Sporns (2009)] in resting-state fMRI data was highly predictive of accurate orientation and that some regions previously associated with allocentric orientation (e.g., hippocampus) were more central within the networks of accurate participants. This suggests that the topological composition of functional networks measured at rest may constrain the degree to which separate brain regions exhibit coherence and integrate information during recollection tasks. Considered together, these findings support the perspective that the degree to which a spatially distributed memory network is able to rapidly exchange information is critical for the effectiveness of its functional output. Importantly, each of these studies identified regions, such as the MTL, that showed increased centrality within the memory network correlating with accurate recall. Thus, due to its centrality within the network of brain areas important to spatial navigation, the MTL specifically may often be identified in fMRI studies attempting to localize brain activity during navigation tasks.

The idea that multiple regions of the brain may interact during spatial representation, with no one area contributing exclusively to allocentric representation, offers a partial reconciliation for seemingly contrasting neuropsychological reports attempting to localize egocentric and allocentric representations to a specific brain region. Specifically, as argued in past reports, networks with similar connectivity and configurations as the brain [termed degenerate networks, see Tononi et al. (1999)] are generally robust to the removal of a single or even random sets of nodes (Albert et al., 2000; Sporns et al., 2004). These data suggest that in some cases the cognitive and behavioral output of a network can be preserved through functional reorganization (Protzner and McAndrews, 2011). In the case of egocentric and allocentric representations, a navigational system in the brain that can dually process both types of representations could theoretically adjust its topological configuration to emphasize processing occurring in preserved cortical regions through increasing their centrality within the functional network active during navigation. This, in turn, might explain why many navigational situations typically involve a dynamic interplay between egocentric and allocentric representations (Byrne et al., 2007; Zhang et al., 2014) rather than the exclusive presence of one or the other.

## THE NETWORK BASIS OF ALLOCENTRIC REPRESENTATION: PREDICTIONS AND LIMITATIONS

It is important that the development of new theoretical models generates directly testable predictions that are falsifiable. We believe that the network perspective, combined with multivariate analytical techniques such as graph theoretical analysis, allow numerous new predictions on the neurocognitive basis of allocentric representation. This may in turn allow some resolution of discrepant findings in past studies and make progress toward understanding such a complex phenomenon. Described below are what we believe to be the most directly applicable, given the large amount of behavioral, neuropsychological, and brain imaging data already available to researchers.

First, if an allocentric representation is indeed primarily dependent on the processing of a distributed network, rather than a single brain region, it is reasonable to assume that lesions affecting the dynamics of this network will impact a person’s ability to solve a spatial task using allocentric information. Although most lesion studies on allocentric representation focus on localized lesions to the hippocampus, as noted above, there is now considerable evidence suggesting both that non-hippocampal lesions can produce allocentric deficits (Habib and Sirigu, 1987; Takahashi et al., 1997; Bohbot et al., 1998; Aguirre and D’Esposito, 1999; Barrash et al., 2000). At the same time, hippocampal lesions often leave some forms of allocentric processing intact (Bohbot et al., 1998; Day et al., 1999; Teng and Squire, 1999; Rosenbaum et al., 2000; Winocur et al., 2005; Bohbot and Corkin, 2007; Winocur et al., 2010). Critically, the network perspective articulated here predicts that the extent to which a lesion disrupts a spatial task involving allocentric processing is dependent upon its impact on the dynamics of the network rather than the removal of a cognitive process generated from the lesioned region (which would be predicted from an aggregate model of brain function). This suggests that lesions in multiple brain regions may influence allocentric processing because numerous spatially distributed regions are co-active during allocentric retrieval tasks (Watrous et al., 2013). This prediction is testable by investigating the effect a lesion has on the topology of the functional network used for allocentric representation, which can be determined using graph theoretical measures quantifying the processing efficiency of the network, as well as dynamic changes in the centrality of different nodes participating in the network following a lesion. Importantly, this leads to a second prediction that navigational ability may be preserved in the case of certain brain lesions given an appropriate rehabilitative context to adjust the types of representations that patients are using to solve navigational tasks (i.e., Caglio et al., 2012). It also supports the perspective that there may not be purely egocentric or allocentric tasks *per se*, as recruitment of a representation type likely isn’t a binary process (i.e., it’s either engaged or not), but rather a question of the relative utility of egocentric and allocentric representations to solve spatial questions about an environment. Preserved allocentric processing may also occur through endogenous network reorganization that shifts the topological configuration of task active functional networks engaged during allocentric spatial tasks to utilize residual functional mechanisms.

Thus, it is reasonable to predict, based on the idea that if network interactions support allocentric representation, conditions associated with the disruption of functional interactions between brain regions may result in deficits in allocentric processing. Recent support for this idea comes from a study on developmental topographical disorientation (DTD), a lifelong cognitive disorder characterized primarily by an inability to encode large scale environmental layouts (Iaria et al., 2009; Iaria and Barton, 2010). In the first group study on the neural basis of DTD, Iaria et al. (2014) found that DTD was associated with aberrant functional connectivity between the right hippocampus and areas within medial and dorsal PFC. These decreases in functional connectivity were apparent without any volumetric differences in brain structure (particularly the hippocampus). These findings suggested that DTD results from a diminished capacity for MTL–PFC functional interactions, which in turn produced the observed deficits in spatial processing and allocentric representation. Although more work needs to be done to describe the neural basis of DTD, these initial results suggest that deficits in allocentric processing may result from changes in how brain regions interact and integrate to form functional networks rather than the computations of one specific brain region.

A second central prediction from this model is that allocentric representation *necessitates* network interactions rather than the activity of a single brain region. This prediction shifts the explanatory emphasis from how a brain region produces a cognitive function toward its role within a network that ultimately results in the cognitive function under investigation. For example, as pointed out previously, if task demands involve integrating multiple locations across different temporal intervals to solve the task, this would likely necessitate the hippocampus. Other situations though, in which locations do not need to be integrated across time yet still involve some relational processing between landmarks, could rely more predominantly on other brain regions, such as PHC and retrosplenial cortex. It is therefore reasonable to speculate that the hippocampus is important for facilitating an exchange of information between regions within a distributed network that needs to be maintained over time intervals, rather than representing spatial information about landmarks *per se*. Indeed, recent findings demonstrating neurons that code for temporal intervals in the hippocampus (Macdonald et al., 2011) as well as the involvement of the hippocampus in spatiotemporal binding (Copara et al., 2014), support this perspective. Importantly, the participation of the hippocampus in spatiotemporal binding also suggests that the topological configuration of task active networks dynamically reorganize their functional connections depending on the type of information needed to be recalled from memory. Evidence for this is provided by Watrous et al. (2013) who found that the strength of pairwise connections between different brain regions in the PFC, MTL, and parietal cortex changed depending on the spatial and temporal nature of the task. In the case of allocentric representations, this model would therefore predict that the degree of temporal interval in which allocentric information needs to be integrated across would correlate with the centrality of the hippocampus within a task active network. Although we are not aware of any study investigating this, it is directly testable using spatiotemporal retrieval tasks that manipulate the temporal interval over which spatial information is acquired and use graph theoretical measures to assess changes in node centrality. Another important test of this prediction is that patients with damage to white matter tracts connecting areas that functionally interact to produce allocentric representation, such as those connecting hippocampus and PFC, with no damage to hippocampus or PFC, should show deficits in allocentric memory. This prediction also remains to be tested.

Finally, despite the potential numerous advantages to a network based model of allocentric representation, it is important to highlight some of its current limitations. First and foremost is how to properly delineate the operation of complex neurocognitive functions such as allocentric representations if they are indeed non-additive. As we have already articulated, we believe the several studies argue against the idea that brain areas contribute additively to allocentric representation. It is unclear, however, how best to operationalize and quantify the precise mechanisms in which an allocentric representation arises through non-linear interactions. As argued by Bassett and Gazzaniga (2011), complex network theory is perhaps best suited for this, although its application to cognitive neuroscience data is relatively new and the analytical techniques to decompose brain networks are developing at a rapid pace. Measures of signal entropy (Costa et al., 2005; Garrett et al., 2013), complexity (Tononi et al., 1998; Deco et al., 2013), dynamic functional connectivity (Zalesky et al., 2014), and graph theory (Rubinov and Sporns, 2011) are promising new approaches designed to capture the richly dynamic and context-dependent basis of information processing in neural networks. A second major limitation is identifying experimental tasks that allow researchers to accurately identify the relative use of allocentric and egocentric representations. As discussed above, despite numerous decades of work, it remains difficult to precisely identify the contribution of each type of representation to a task, suggesting that a better strategy may be to develop an allocentric/egocentric index. Such work has recently been carried out by Marchette et al. (2011) in which they quantified an individuals tendency to utilize either an allocentric or egocentric representation. Using a continuous variable to measure response strategy would facilitate decomposing the dynamics of memory networks both within and between subjects.

## FINAL NOTE: HIPPOCAMPAL PLACE CELLS AS AN EXAMPLE OF MODULAR CODING OF THE ALLOCENTRIC REPRESENTATION?

Thus far, we have focused on behavioral, lesion, and fMRI studies, which argue against allocentric navigational strategies depending on a single brain region (**Figure 3A**) and as decomposable into contributions from individual brain regions (**Figure 3B**). One might argue, as others have (O’Keefe and Nadel, 1978; Redish, 1999), however, that place cells, present in the rodent, monkey, and human hippocampus (O’Keefe and Dostrovsky, 1971; Ekstrom et al., 2003; Hori et al., 2003), are the neural instantiation of an allocentric representation, or cognitive map. While place cells do have many features similar to what one might expect in a neural systems that code spatial environments in a map-like fashion, there are other important features of place cells that are decidedly not map-like. Place cells in the rodent and human hippocampus remap based on egocentric direction (Markus et al., 1995; Miller et al., 2013), are sensitive to goal and other temporal variables (Gothard et al., 2001; Hollup et al., 2001; Ekstrom et al., 2003; Bahar et al., 2011), and remap with subtle changes to the spatial geometry of the environment (Leutgeb et al., 2005; Wills et al., 2005). Indeed, recent theoretical models of the cognitive map now suggest that time and geometry less variant spatial coding mechanisms possibly resides outside of the hippocampus (Buzsaki, 2006; Buzsaki and Moser, 2013). Grid cells, neurons in enthorhinal cortex that fire in a regularly spaced fashion as the rat explores a spatial environment (Fyhn et al., 2004; Jacobs et al., 2013), may be a better candidate for the neural basis of allocentric representation (Buzsaki and Moser, 2013). Yet lesions of entorhinal cortex, at least in rodents, do not abolish place cell firing in the CA3 subfield of the hippocampus (Lu et al., 2013) and impair, but do not abolish, the place code in CA1 (Brun et al., 2008). While many details of entorhinal–hippocampal neural interactions remain to be established, grid cells do not contribute in a clear or modular fashion to place coding in the hippocampus, at least based on what the above-mentioned studies have determined so far in the rat. Furthermore, in addition to grid cells, entorhinal cortex cells also respond to egocentric direction (Sargolini et al., 2006), suggesting this area may not be specialized for allocentric computations alone. In addition, consistent with what we have argued here, it is clear that other areas, like prefrontal and retrosplenial cortex, also contribute critically, via oscillatory synchrony, to spatial coding in the hippocampus (Benchenane et al., 2010; Battaglia et al., 2011; Fujisawa and Buzsaki, 2011). Thus, although many aspects of the hippocampal neural code would appear sufficient to support an allocentric representation, the neural code itself is not map-like and depends, at least in part, on coordinated input and activity from other brain structures.

## CONCLUDING REMARKS

We began our discussion by pointing out some of the difficulties in pinning down the exact nature of the allocentric representation. In many situations in which one might expect a “pure” allocentric representation to predominate, such as reasoning about distances on a map or using short-cuts while navigating, egocentric representations bias how we utilize an allocentric representation. In fact, while we identified situations in which an allocentric representation might dominate, such as when participants make judgments involving relative distances or directions of objects to each other, egocentric representations still serve as important anchors and cues in solving these tasks. Even individuals may vary in the degree to which they utilize a primarily egocentric or allocentric strategy to solve a task, and even within individuals use of these strategies may vary during a task. Our inability to identify “process-pure” allocentric tasks suggested that it might also be difficult to unambiguously identify situations in which lesions to one brain region abolish allocentric memory. This appears to be particularly true in humans, in which multiple brain appear necessary for situations involving allocentric computations, including PHC, retrosplenial cortex, PFC, and hippocampus. The lack of one brain region as central to navigation involving allocentric computations suggested the possibility that this behavior might be better described as a network phenomenon. We discussed two different perspectives on this issue, both of which remain to be fully tested. One view, the additive model, argues that an allocentric representation emerges due to additive computations from individual brain regions. A different perspective, that we advocate here, is that an allocentric representation emerges from non-additive computations shared across multiple interacting brain regions. We concluded with several predictions related to spatial memory tasks that provide critical tests of the non-additive network model vs. the specialized perspective more frequently adopted in the literature.

## Conflict of Interest Statement

The authors declare that the research was conducted in the absence of any commercial or financial relationships that could be construed as a potential conflict of interest.
